# Perioperative high density lipoproteins, oxidative stress, and kidney injury after cardiac surgery

**DOI:** 10.1016/j.jlr.2021.100024

**Published:** 2021-01-14

**Authors:** Loren E. Smith, Derek K. Smith, Patricia G. Yancey, Valentina Kon, Alan T. Remaley, Frederic T. Billings, MacRae F. Linton

**Affiliations:** 1 Department of Anesthesiology, Vanderbilt University Medical Center, Nashville, TN, USA; 2 Department of Biostatistics, Vanderbilt University Medical Center, Nashville, TN, USA; 3 Department of Medicine, Vanderbilt University Medical Center, Nashville, TN, USA; 4 Department of Pediatrics, Vanderbilt University Medical Center, Nashville, TN, USA; 5 National Institutes of Health, National Heart, Lung, and Blood Institute, Bethesda, MD, USA

**Keywords:** cardiovascular disease, cholesterol, eicosanoids, HDL function, paraoxonase, renal disease

## Abstract

Oxidative stress promotes acute kidney injury (AKI). Higher HDL cholesterol concentrations are associated with less AKI. To test the hypothesis that HDL antioxidant activity is associated with AKI after cardiac surgery, we quantified HDL particle (HDL-P) size and number, paraoxonase-1 (PON-1) activity, and isofuran concentrations in 75 patients who developed AKI and 75 matched control patients. Higher preoperative HDL-P was associated with less AKI (OR: 0.80; 95% CI, 0.71–0.91; *P* = 0.001), higher PON-1 activity ( *P* < 0.001), and lower plasma concentrations of isofurans immediately after surgery (*P* = 0.02). Similarly, higher preoperative small HDL-P was associated with less AKI, higher PON-1 activity, and lower isofuran concentrations. Higher intraoperative particle losses were associated with less AKI (OR: 0.79; 95% CI 0.67–0.93; *P* = 0.005), and with decreased postoperative isofuran concentrations (*P* = 0.04) . Additionally, higher preoperative small HDL-P and increased intraoperative small particle loss were associated with improved long-term renal function (*P* = 0.003, 0.01, respectively). In conclusion, a higher preoperative concentration of HDL-P, particularly small particles, is associated with lower oxidative damage and less AKI. Perioperative changes in HDL-P concentrations are also associated with AKI. Small HDL-P may represent a novel modifiable risk factor for AKI.

Acute kidney injury (AKI) after cardiac surgery occurs in up to 30% of patients and is independently associated with increased hospital length of stay, postoperative atrial fibrillation, and death (1, 2, 3). Additionally, patients who develop and fully recover from AKI after cardiac surgery have an increased risk of developing chronic kidney disease within weeks to months of surgery compared with patients without postoperative AKI (4). Current treatments for AKI are limited, and novel therapeutic targets for the prevention or treatment of postoperative AKI are needed.

Cardiac surgery patients with higher preoperative HDL cholesterol (HDL-C) concentrations are less likely to develop AKI, independent of other AKI risk factors (5). Oxidative stress is an important mechanism in the pathogenesis of postoperative AKI, and higher concentrations of F2-isoprostanes and isofurans, in vivo products of lipid peroxidation, are associated with an increased risk of AKI after cardiac surgery (6, 7, 8, 9). The primary HDL-associated antioxidant enzyme, paraoxonase-1 (PON-1), is enriched on small, dense HDL particles (HDL-P). PON-1 reduces oxidized phospholipids, including F2-isoprostane and isofuran precursors, and inhibits the prooxidant enzyme, myeloperoxidase (10, 11, 12). The impact of HDL PON-1 activity on postoperative AKI is unknown.

While previous studies have examined the association between HDL-C and postoperative AKI, HDL-P concentration is more strongly associated with cardiovascular disease risk and may represent HDL function better than HDL-C (13, 14). To test the hypothesis that HDL-P and HDL antioxidant capacity are associated with oxidative damage during surgery and postoperative AKI, we quantified and compared small, medium, large, and total HDL-P concentrations and PON-1 activity throughout the perioperative period and the nonesterified isofuran concentrations directly after surgery in 75 cardiac surgery patients who developed AKI and 75 matched control patients.

## Materials and methods

### Patients

We tested this hypothesis in a case-control study nested within the Statin AKI Cardiac Surgery RCT, a randomized trial of atorvastatin to prevent postoperative AKI (NCT00791648) (15). During this trial, perioperative oxidative stress was prospectively characterized. There was no difference in preoperative HDL-C concentrations between long-term statin using and statin naïve patients or between atorvastatin and placebo treatment groups. Further, atorvastatin treatment did not affect AKI. The trial included patients who underwent major elective cardiac surgery requiring thoracotomy or sternotomy at a tertiary academic medical center. Patients with liver dysfunction, acute coronary syndrome, history of kidney transplant, or preoperative renal replacement therapy, pregnancy, or statin intolerance were excluded. We selected 75 patients who developed AKI and 75 patients who did not develop AKI, matched by the Thakar risk score for severe AKI after cardiac surgery and cardiopulmonary bypass exposure time (16). The Thakar score accounts for gender, preoperative serum creatinine concentration, insulin-dependent diabetes, congestive heart failure, and type of cardiac surgery. AKI was defined using the Acute Kidney Injury Network serum creatinine criteria over the first 48 postoperative hours (17). This study was conducted according to the standards of the Declaration of Helsinki after approval by our institutional review board. All participants provided written informed consent.

### Data and sample collection

All patient demographic and clinical data were prospectively collected by trained research staff. Serum creatinine concentration was measured in a certified clinical laboratory before surgery and on the mornings of postoperative days 1 and 2 and were complete, and >99% of all demographic data were complete. Plasma and serum samples for laboratory analysis were collected at induction of anesthesia, at admission to the intensive care unit directly after surgery, and at 9:00am on postoperative day 2, centrifuged at 1,000*g* for 15 min, and frozen at −80°C until analyte measurement (no freeze-thaw cycles).

Because postoperative AKI predicts subsequent chronic renal dysfunction (4), the association between perioperative HDL-P and long-term serum creatinine concentration was also examined. We defined long-term serum creatinine concentration as creatinine measured 3–12 months after surgery. If creatinine was measured multiple times during this period, the measurement made closest to 12 months after surgery was collected. Eighty-six patients in the study cohort, 43 patients who developed postoperative AKI and 43 control patients, had long-term serum creatinine measurements available.

### Specimen analyses

Serum creatinine, triglyceride, total cholesterol, and low-density lipoprotein cholesterol concentrations were measured in the hospital’s clinical laboratory on an Abbott ACHITECT platform, Clinical Chemistry model (Abbott Park, Illinois). HDL-P and HDL-C concentrations were measured at the National Institutes of Health Lipoprotein Metabolism laboratory (Bethesda, MD) using the NMR-based Lipoprofile test on a Vantera Clinical Analyzer utilizing the LP4 algorithm (LabCorp, Burlington, North Carolina).

Nonesterified, non-HDL–associated, isofuran concentrations were measured in plasma using gas chromatography-mass spectrometry (18). Isofurans are end-products of nonenzymatic arachidonic acid peroxidation that are stable in vivo, increase in proportion to increasing oxidative stress, and have been independently associated with perioperative organ injury (7, 19). To estimate the antioxidant activity of HDL, we measured PON-1 activity in equal volumes of apolipoprotein B-depleted serum using the fluorescent substrate 7-diethylphospho-6,8-dixuor-4-methylumbelliferyl (20). PON-1 can hydrolyze oxidized phospholipids, aromatic carboxylic acid esters, and organophosphates and is found primarily on HDL-P (21). PON-1 activity was measured in duplicate and normalized to a standard apolipoprotein B-depleted serum sample derived from six healthy volunteers.

### Statistical analyses

Demographic, laboratory, and clinical data were summarized as n (%) for binary variables and 50th (10th, 90th) percentile for continuous variables. Two-tailed Student’s t-tests were used to compare HDL-P between timepoints. The correlations between HDL-P and PON-1 activity, intraoperative HDL-P loss, volume of intraoperative fluid administered, length of surgery, and markers of preoperative kidney function were estimated with Pearson’s correlation coefficients. Longitudinal HDL-P changes between patients who did and did not develop AKI were compared using a mixed effects model. Multivariate logistic and linear regression models were used to estimate the associations between HDL-P concentrations and changes in HDL-P between timepoints and AKI, preoperative and postoperative plasma isofuran concentrations, and long-term serum creatinine concentration. Multivariate logistic and linear regression models were also used to estimate the associations between triglyceride-rich lipoprotein particle changes, AKI, and postoperative plasma isofuran concentrations. To ensure our primary analysis of the association between HDL-P and AKI was not confounded by inequalities in our patient groups, we calculated a propensity score accounting for imbalances in age, sex, body mass index, baseline estimated glomerular filtration rate (eGFR), hypertension, and diabetes and reassessed the association between HDL-P and AKI with propensity score adjustment. An interaction term was used to determine if preoperative HDL PON-1 activity modified the association between intraoperative HDL-P loss and AKI. Covariates for triglyceride-rich lipoprotein-P, HDL-P, and HDL-P size models included age, gender, preoperative eGFR, and history of diabetes mellitus. For models assessing intraoperative triglyceride-rich lipoprotein particle and HDL-P change, volume of administered intraoperative fluid, including red blood cell transfusion volume, was also included. Models of postoperative isofuran concentrations were adjusted for body mass index and volume of administered intraoperative fluid. All covariates were selected a priori. All analyses were performed using Stata software, version 15.1. A type-I error rate of 5% determined statistical significance for all analyses.

## Results

### Study participants

The median (10th, 90th percentile) age of the patient sample was 69 years (50, 82), and 30% of patients were female (Table 1). Among patients who developed postoperative AKI, the median preoperative HDL-C concentration was 38 mg/dL (30, 50), and HDL-P was 15 μmol/L (10, 18). Among control patients, the median preoperative HDL-C concentration was 40 mg/dL (33, 62), and HDL-P was 17 μmol/L (13, 21). The increase in serum creatinine from baseline to postoperative day 1 in the AKI group was 0.19 mg/dL (−0.10, 0.62) and was −0.07 mg/dL (−0.29, 0.11) in the non-AKI group. The increase in serum creatinine from baseline to postoperative day 2 in the AKI group was 0.59 mg/dL (0.35, 1.09) and was −0.02 mg/dL (−0.21, 0.16) in the non-AKI group. Median baseline serum creatinine concentration was 1.20 mg/dL (0.77, 1.90) in the AKI group and 1.12 mg/dL (0.83, 1.60) in the non-AKI group. Rates of congestive heart failure, previous myocardial infarction, and peripheral vascular disease were similar between groups. More patients in the AKI group had diabetes mellitus compared with the non-AKI group. Median Thakar risk score was 3 (1, 4) in the AKI group and 3 (1, 4) in the non-AKI group. The median cardiopulmonary bypass exposure time was 125 min (0, 258) in the AKI group and 122 min (0, 211) in the non-AKI group.

**Table 1:** Study cohort characteristics

Characteristic^a^	Non-AKI Patients	AKI Patients	*P* value
Age	66 (47, 81)	71 (53, 82)	0.03
Female	28 (37%)	17 (23%)	0.07
Caucasian	71 (95%)	71 (95%)	1
Body mass index (kg/m^2^)	28 (23, 35)	30 (24, 40)	0.01
Medical history			
Diabetes mellitus	14 (19%)	35 (47%)	<0.001
Hypertension	64 (85%)	73 (97%)	0.03
Myocardial infarct	11 (15%)	13 (17%)	0.91
Congestive heart failure	32 (43%)	42 (56%)	0.21
Peripheral vascular disease	21 (28%)	28 (37%)	0.29
Current smoker	13 (17%)	7 (9%)	0.28
Chronic statin use	47 (63%)	57 (76%)	0.15
Baseline laboratory data			
Total cholesterol (mg/dL)	131 (98, 160)	129 (94, 179)	0.72
High density lipoprotein cholesterol (mg/dL)	36 (26, 58)	35 (25, 50)	0.47
Low density lipoprotein cholesterol (mg/dL)	71 (44, 104)	68 (43, 111)	0.13
Triglycerides (mg/dL)	95 (54, 179)	94 (51, 196)	0.42
eGFR (mL/min/1.73 m^2^)	66 (40, 92)	54 (35, 86)	0.03
Creatinine (mg/dL)	1.12 (0.83, 1.59)	1.20 (0.80, 1.87)	0.01
Intraoperative events			
Cardiopulmonary bypass use	68 (91%)	57 (76%)	0.02
Cardiopulmonary bypass duration (min)	125 (77, 211)	122 (0, 258)	0.81
Intraoperative red blood cell transfusion (units)	0 (0, 4)	1 (0, 7)	0.05
Outcomes			
Acute kidney injury at 48 h			
Any stage	0 (0%)	75 (100%)	
Stage 1	0 (0%)	66 (88%)	
Stage 2	0 (0%)	4 (5%)	
Stage 3	0 (0%)	5 (7%)	
48 h creatinine rise from baseline (mg/dL)	−0.02 (−0.21, 0.16)	0.59 (0.35, 1.09)	
Dialysis, postoperative	0 (0%)	2 (3%)	
Plasma isofurans (pg/mL) after surgery	70 (39, 124)	86 (43, 158)	0.13
CKMB, postoperative day 1 (mg/dL)	25 (10, 98)	28 (6, 65)	0.18
TIA or stroke	4 (5%)	5 (7%)	0.98
Length of stay, days	7 (5, 11)	9 (6, 18)	<0.001
Serum creatinine at long-term follow-up (mg/dL)	1.13 (0.83, 1.72)	1.51 (0.85, 2.51)	0.02

AKI, acute kidney injury.

a Continuous variables are reported as 50th (10th, 90th) percentile, and binary variables are reported as n (%). CABG, coronary artery bypass graft; CKMB, creatine kinase myocardial band; eGFR, estimated glomerular filtration rate; TIA, transient ischemic attack.

### HDL particles during the perioperative period

**Fig. 1:**
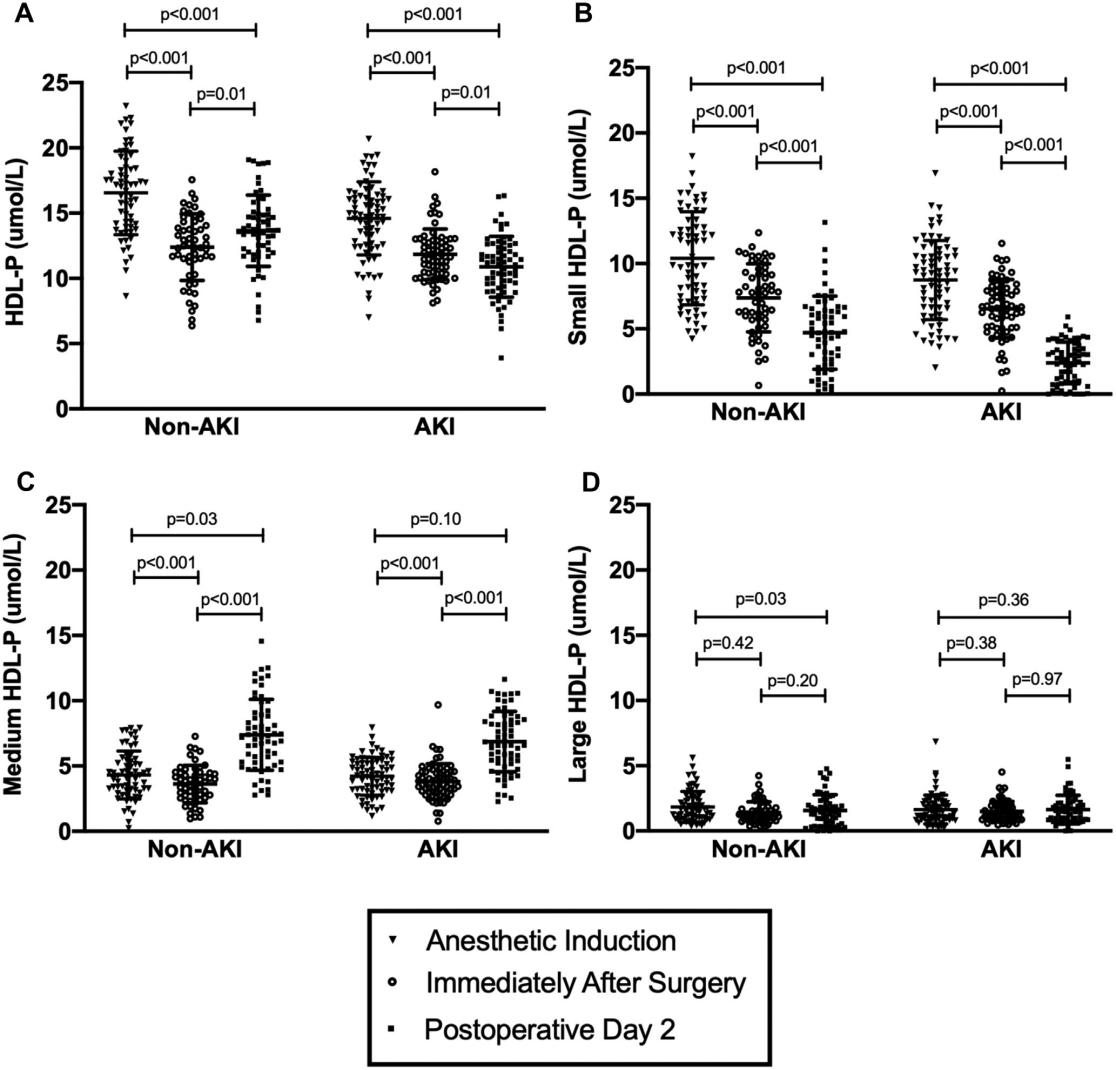
HDL particle concentrations during the perioperative period. A: total HDL particles, (B) small HDL particles, (C) medium HDL particles, (D) large HDL particles. Bars represent the mean ± one standard deviation. N = 150. AKI, acute kidney injury; HDL, high density lipoprotein.

In both the AKI and non-AKI groups, HDL-P decreased intraoperatively and remained below preoperative levels throughout the first 48 postoperative hours (Fig. 1A). Medium and small HDL-P decreased intraoperatively in both groups, with the greatest decrease in small HDL-P populations (Fig. 1B–D). The intraoperative decrease in HDL-P was not correlated with the total volume of intravenous fluid and blood administered during surgery (R = 0.03, *P* = 0.72) or with the length of surgery (R = −0.13, *P* = 0.17). The magnitude of intraoperative and postoperative HDL-P losses were also not correlated with preoperative eGFR (R = 0.10, −0.02, *P* = 0.28, 0.84, respectively) or preoperative urine albumin-to-creatinine ratio (R = 0.08, 0.18, *P* = 0.55, 0.19, respectively), two markers of preoperative kidney function. Additionally, the median intraoperative HDL-P loss in patients exposed to cardiopulmonary bypass was 3.1 μmol/L, whereas the median intraoperative HDL-P loss in patients not exposed to cardiopulmonary bypass was 3.9 μmol/L.

### HDL particles and postoperative AKI

**Fig. 2:**
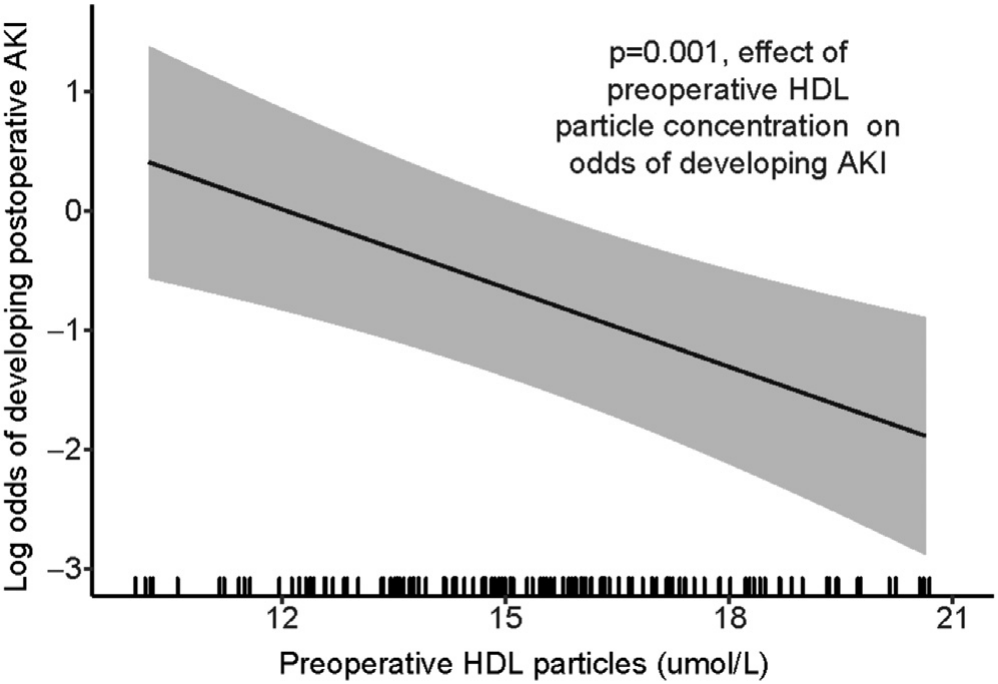
Partial effect plot showing the association between preoperative HDL particle concentration and the odds of developing postoperative AKI, adjusted for model covariates. The x-axis rug plot displays the distribution of subjects. Shading designates the 95% confidence interval. AKI, acute kidney injury; HDL, high density lipoprotein.

After adjustment for AKI risk factors, preoperative HDL-C was not independently associated with the odds of developing postoperative AKI (OR, 0.97; 95% CI, 0.93–1.003; *P* = 0.07). In contrast, a higher preoperative HDL-P was associated with lower odds of developing postoperative AKI (OR, 0.80; 95% CI, 0.71–0.91; *P* = 0.001, Fig. 2). Propensity score analysis accounting for inequality in patient groups did not meaningfully change the association between preoperative HDL-P and AKI (OR 0.81; 95% CI, 0.70–0.93; *P* = 0.002). Higher preoperative concentrations of small HDL-P were also associated with lower odds of developing AKI (OR, 0.84; 95% CI, 0.75–0.94; *P* = 0.002), whereas preoperative concentrations of medium and large HDL-P were not associated with AKI (*P* = 0.75 and 0.48, respectively).

**Fig. 3:**
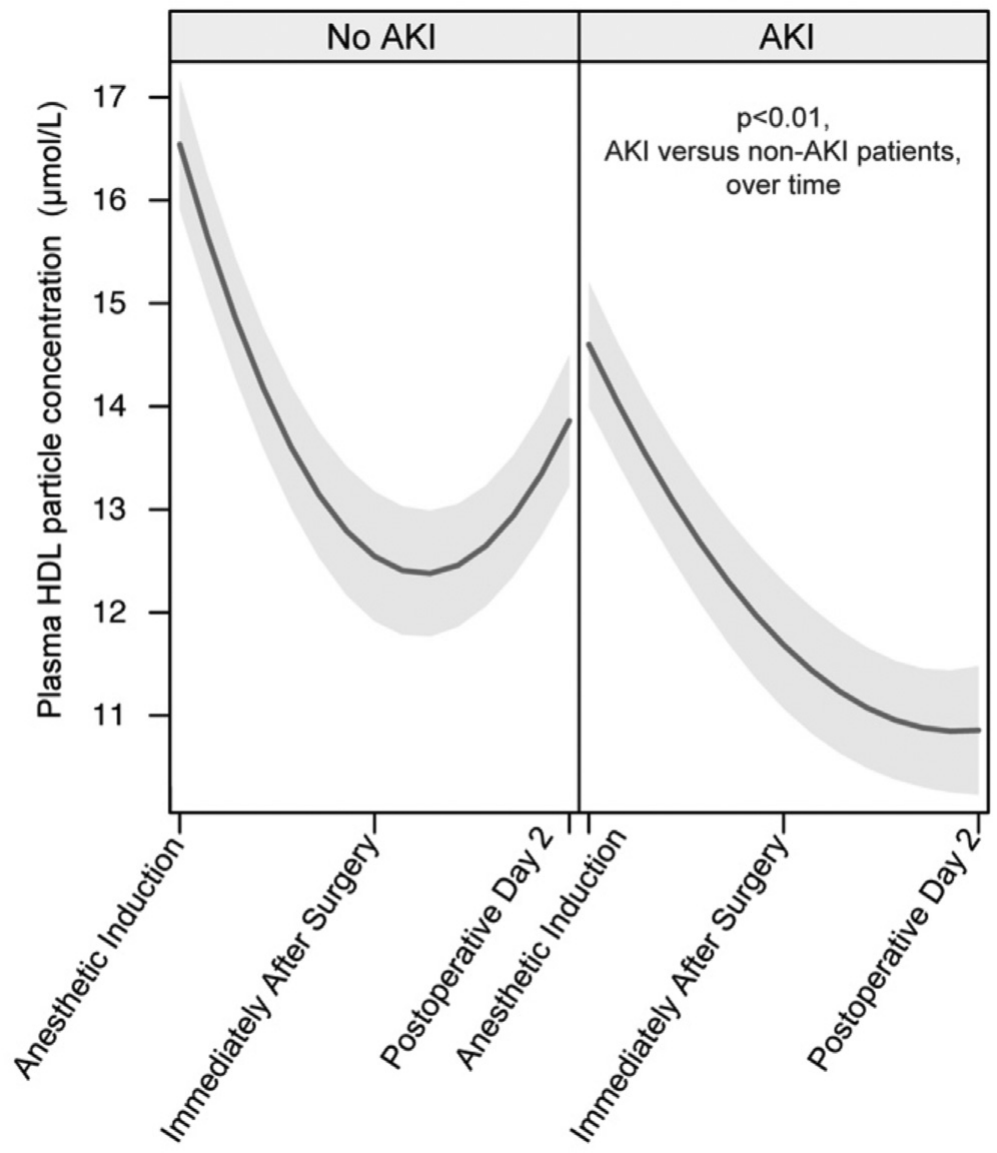
Plot of HDL particle concentration estimates during the perioperative period based on AKI status. Shading designates the 95% confidence interval. AKI, acute kidney injury; HDL, high density lipoprotein.

Next, we examined the association between circulating concentrations of HDL-P during the intraoperative period and AKI. Patients who did not develop AKI lost more HDL-P intraoperatively but regained HDL-P in the circulation over the first 48 postoperative hours. In contrast, patients who developed AKI lost less HDL-P intraoperatively but continued to lose HDL-P during the first 48 postoperative hours (*P* = 0.009, Fig. 3). After adjustment for AKI risk factors, higher intraoperative HDL-P loss from the circulation was associated with lower odds of developing AKI (OR, 0.79; 95% CI, 0.67–0.93; *P* = 0.005). The association between HDL-P loss during surgery and AKI was stronger than the association between HDL-C loss during surgery and AKI (OR 0.93; 95% CI 0.87–0.98; *P* = 0.01). The association between HDL-P loss and AKI was not specific to HDL size. Further, there was no association between HDL-P immediately after surgery and AKI (*P* = 0.60).

In contrast, postoperative HDL-P loss was directly associated with the odds of developing AKI (OR, 1.91; 95% CI, 1.43–2.53; *P* < 0.001), as was HDL-P concentration measured on postoperative day 2 (OR 0.65; 95% CI, 0.53–0.79; *P* < 0.001). Again, the association between postoperative HDL-P loss and AKI was stronger than the association between postoperative HDL-C loss and AKI (OR 1.10; 95% CI 1.01–1.20; *P* = 0.02). Loss of small HDL-P and medium HDL-P during the postoperative period was also associated with the odds of developing AKI (small particle loss OR 1.24; 95% CI, 1.10–1.52; *P* = 0.04, medium particle loss OR 1.20; 95% CI, 1.03–1.41; *P* = 0.02). Postoperative large HDL-P loss was not associated with the odds of AKI (*P* = 0.47).

To confirm that perioperative plasma volume changes do not account for the associations between HDL-P loss and AKI reported above, the associations between triglyceride-rich lipoprotein particle loss, which would be subject to the same perioperative changes in plasma volume as HDL, and AKI was also examined. Neither the magnitude of intraoperative triglyceride-rich lipoprotein particle loss from the circulation nor the magnitude of postoperative triglyceride-rich lipoprotein particle loss was associated with the odds of developing AKI (*P* = 0.39, 0.07 respectively).

### HDL particles and oxidative damage during surgery

Higher preoperative HDL-P correlated with higher preoperative PON-1 antioxidant activity (R = 0.36, *P* < 0.001). Higher preoperative concentrations of small HDL-P were similarly correlated with higher preoperative PON-1 activity (R = 0.30, *P* = 0.002), whereas medium and large HDL-P concentrations were not correlated with PON-1 activity (*P* = 0.59, 0.66, respectively). Higher preoperative concentrations of small HDL-P were similarly correlated with higher preoperative PON-1 activity (R = 0.30, *P* = 0.002), whereas medium and large HDL-P concentrations were not correlated with PON-1 activity (*P* = 0.59, 0.66, respectively). Higher preoperative PON-1 activity was not associated with the odds of developing AKI (*P* = 0.29). Further, on average, HDL-associated PON-1 activity in the circulation did not significantly change during the intraoperative period or over the first 2 postoperative days (*P* = 0.58 and 0.89, respectively). There was no association between the PON-1 activity level associated with the HDL-P which remained in the circulation directly after surgery and AKI (*P* = 0.08). There was also no association between the PON-1 activity level associated with HDL-P in the circulation 2 days after surgery and AKI (*P* = 0.66). However, PON-1 activity loss during surgery was associated with HDL-P loss during surgery (*P* < 0.001) even after adjustment for volume of intraoperative fluid and blood administered during surgery. Further, a higher preoperative HDL PON-1 antioxidant activity modified the effect of HDL-P loss on AKI (*P* = 0.006). Specifically, if a patient’s preoperative HDL PON-1 activity was low (25th percentile), a 1 μmol/L intraoperative decrease in HDL-P was associated with an absolute risk reduction of developing AKI of 2%. If a patient’s preoperative HDL PON-1 activity level was high (75th percentile), a 1 μmol/L intraoperative decrease in HDL-P was associated with an absolute risk reduction of developing AKI of 13%. PON-1 activity loss during the first 48 h after surgery was not associated with HDL-P loss during this time period (*P* = 0.08).

**Fig. 4:**
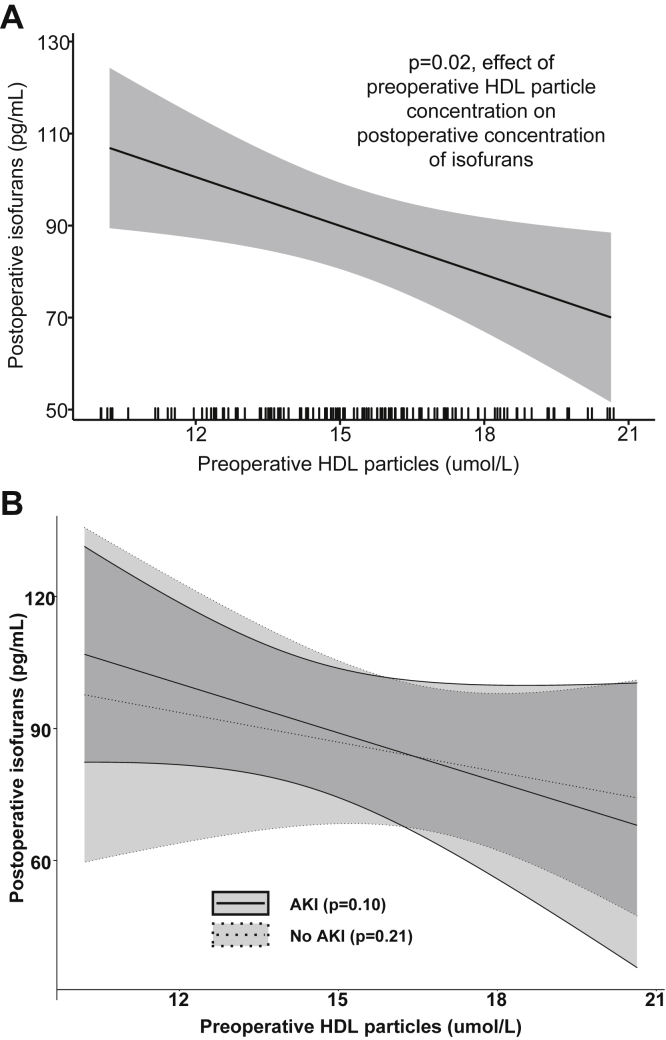
Partial effect plots showing the association between preoperative HDL particle concentration and postoperative isofuran concentrations, adjusted for model covariates. A: total cohort, (B) cohort divided by AKI status. The x-axis rug plot in panel A displays the distribution of subjects. Shading designates the 95% confidence interval. AKI, acute kidney injury; HDL, high density lipoprotein.

Next, we tested the hypothesis that higher HDL-P concentrations were associated with less in vivo oxidative damage during surgery. A higher preoperative HDL-P was independently associated with a lower plasma concentration of isofurans immediately after surgery (*P* = 0.02, Fig. 4A), such that every 1 μmol/L increase in preoperative HDL-P was associated with a 3.5 pg/mL decrease in postoperative plasma isofuran concentration. Similarly, a higher preoperative concentration of small HDL-P was independently associated with a lower plasma concentrations of isofurans immediately after surgery (*P* = 0.007), such that every 1 μmol/L increase in preoperative small HDL-P was associated with a 3.6 pg/mL decrease in postoperative plasma isofuran concentration. Preoperative concentrations of medium and large HDL-P were not associated with postoperative isofuran concentrations (*P* = 0.16, 0.25, respectively). Further, when the cohort was analyzed based on AKI status, preoperative HDL-P concentration showed a statistically nonsignificant trend toward being inversely related to postoperative nonesterified isofuran concentration in both the AKI and non-AKI subgroups, with a similar effect size observed in each subgroup as was seen when the groups were analyzed together (*P* = 0.10, 0.21 respectively, Fig. 4B).

Greater intraoperative HDL-P loss was also independently associated with lower plasma isofuran concentrations immediately after surgery (*P* = 0.04). Specifically, a 1 μmol/L intraoperative loss of HDL-P from the circulation was associated with a 3.4 pg/mL decrease in plasma isofurans. In contrast, intraoperative triglyceride-rich lipoprotein particle loss was not associated with plasma isofuran concentrations immediately after surgery (*P* = 0.22).

### Perioperative HDL particles and long-term kidney function

As a sensitivity analysis, we examined the association between perioperative HDL-P concentrations and serum creatinine concentration measured 3–12 months after surgery. In patients who developed AKI, the median serum creatinine concentration at long-term follow-up was 1.51 mg/dL (0.85, 2.51), whereas in patients who did not develop AKI, the median serum creatinine concentration at long-term follow-up was 1.13 mg/dL (0.83, 1.72). There was no association between preoperative HDL-P and long-term serum creatinine concentrations after surgery (*P* = 0.22). In contrast, a higher preoperative small HDL-P concentration was independently associated with a lower long-term serum creatinine concentration after surgery (*P* = 0.003). Each 1 μmol/L increase in preoperative small HDL-P was associated with a 0.08 mg/dL decrease in long-term serum creatinine concentration after surgery.

Increased intraoperative HDL-P loss was also associated with lower long-term serum creatinine after surgery (*P* = 0.01). Each 1 μmol/L decrease in intraoperative HDL-P was associated with a 0.05 mg/dL decrease in long-term serum creatinine concentration after surgery. Increased intraoperative small HDL-P loss was also associated with a lower long-term serum creatinine concentration after surgery (*P* = 0.04), such that each 1 μmol/L intraoperative decrease in small HDL-P was associated with a 0.11 mg/dL decrease in long-term serum creatinine concentration. No association was found between intraoperative loss of medium or large HDL-P or postoperative HDL-P losses and long-term serum creatinine concentrations (*P* = 0.45, 0.62, and 0.34, respectively).

## Discussion

Increased concentrations of HDL-P and particularly small HDL-P were associated with higher PON-1 activity, lower postoperative isofuran concentrations, and lower odds of AKI after cardiac surgery. In both AKI and non-AKI groups of patients, a significant portion of HDL-P, particularly small HDL-P, was lost from the circulation during the perioperative period, but the pattern of particle loss was different in patients who did and did not develop AKI. Patients who did not develop AKI lost more HDL-P intraoperatively but regained more HDL-P over the first 48 postoperative hours, whereas AKI patients lost more HDL-P from the circulation postoperatively. Higher intraoperative HDL-P loss was also independently associated with lower odds of developing postoperative AKI and decreased systemic oxidative stress. In contrast, higher postoperative HDL-P loss was independently associated with greater odds of developing AKI. Further, higher preoperative small HDL-P concentrations and higher intraoperative HDL-P loss were associated with improved long-term renal function.

PON-1 is exclusively carried on HDL-P, and small HDL-P preferentially bind PON-1 and exhibit potent antioxidant properties (22, 23, 24). In patients undergoing diagnostic coronary angiography, higher PON-1 activity has been associated with lower oxidative damage biomarkers including lower isoprostane concentrations and a decreased long-term risk of major adverse cardiac events (25). The current study is the first to demonstrate an association between higher concentrations of antioxidant HDL, reduced oxidative stress during surgery, and less postoperative kidney injury. Our data suggest that HDL-P and HDL antioxidant activity may impact postoperative AKI. Additionally, pharmacological treatments which acutely increase small antioxidant HDL-P are currently under development and clinical testing, such as the reconstituted HDL medication CSL-112 (26). It is possible these medications could be tested in perioperative trials to reduce postoperative AKI.

In the current study, we also observed a rapid intraoperative decrease in HDL-P and that a pattern of prolonged particle loss *versus* recovery was associated with AKI. Rapidly decreasing HDL has been observed in other critically ill patient populations, and a pattern of prolonged HDL decline *versus* recovery has been associated with in-hospital death (27, 28). Surprisingly, in cardiac surgery patients, intraoperative HDL-P losses from the circulation were associated with lower odds of AKI. Our data cannot determine why intraoperative particle loss is associated with less AKI, but we offer several possibilities below.

HDL can become dysfunctional and proinflammatory during cardiac surgery, and loss of dysfunctional particles might preserve renal function (29). Increased HDL catabolism for cellular repair or steroid synthesis might also preserve renal function. In this context, continued postoperative HDL-P decline might result from continued or unresolved renal damage necessitating continued HDL catabolism. Alternatively, because the majority of HDL-P lost were small particles that can readily enter the perivascular and renal interstitial spaces, intraoperative HDL-P loss may be because of increased particle migration out of the circulation (30, 31, 32, 33, 34, 35). In the interstitial space, surgically induced reactive oxygen species promote lipid peroxidation and isofuran formation, which promote AKI (6, 7). In our study, higher intraoperative particle losses were associated with lower postoperative isofuran concentrations and lower odds of AKI, particularly in patients with high HDL antioxidant PON-1 activity. Under homeostatic conditions, 50% of HDL resides within interstitial spaces, where it takes approximately 29 h to return to the circulation (36, 37). It is unknown if HDL flux through the interstitium changes during surgery. It is also unclear if the HDL-P increase seen postoperatively in patients that did not develop AKI represents a net positive flux of HDL from the interstitial space back into the circulation, an increased synthesis of HDL, a decreased catabolism of HDL, or decreased renal excretion of HDL due to preserved renal function. Further, it is unclear if the continued postoperative particle loss seen in patients that developed AKI is because of renal loss of HDL and is a consequence of perioperative renal damage.

This study has several limitations. Because we cannot randomize surgical patients to higher or lower preoperative HDL-P concentrations or to higher or lower intraoperative HDL-P losses, our data cannot support causative conclusions. Moreover, while we matched our AKI and control patients using preoperative and intraoperative AKI risk factors, differences between the groups remain. For example, more diabetic patients were present in the AKI group. To minimize the risk of confounding, analyses were adjusted for age, gender, preoperative eGFR, and diabetes. Further, we performed a sensitivity analysis with a propensity score accounting for age, sex, body mass index, baseline eGFR, and a past medical history of hypertension and diabetes. This propensity score adjustment resulted in no appreciable change in the association between HDL-P and AKI, suggesting our standard model adjustments for age, gender, preoperative eGFR, and diabetic status were adequate.

In conclusion, higher concentrations of HDL-P and small HDL-P were associated with lower systemic oxidative stress, lower odds of AKI, and improved long-term renal function after cardiac surgery. Further, the pattern of perioperative HDL-P loss and recovery differed between patients who did and did not develop AKI, and perioperative changes in HDL-P concentration in the circulation were independently associated with AKI. Future studies characterizing the effects of synthetic HDL on acute renal injury and tracing HDL-P during the perioperative period are needed to develop a better understanding of HDL metabolism and function during acute stress and to elucidate the roles HDL may have in perioperative renal protection.

### Data availability

Data that support the findings of this study are available from the corresponding author upon reasonable request.

## Conflict of interest

L. E. S., D. K. S., P. G. Y., V. K., A. T. R., and F. T. B. report no conflicts. M. F. L. has received grant support from Regeneron, Sanofi, Merck, Ionis, and PCORI, and consulting fees from AMGEN and REGENXBIO.
